# Comparative Assessment of Factors Involved in Acetoin Synthesis by *Bacillus subtilis* 168

**DOI:** 10.1155/2014/578682

**Published:** 2014-03-10

**Authors:** Pratibha Sharma, Santosh Noronha

**Affiliations:** ^1^Department of Bioscience and Bioengineering, Indian Institute of Technology Bombay, Powai, Mumbai 400076, India; ^2^Department of Chemical Engineering, Indian Institute of Technology Bombay, Powai, Mumbai 400076, India

## Abstract

Acetoin is widely used as flavor agent and serves as a precursor for chemical synthesis. Here we focused on identifying the best physiological conditions (initial substrate concentrations, pH, temperature, and agitation) for enhanced acetoin accumulation by *Bacillus subtilis* 168. The optimal physiological conditions support maximum acetoin accumulation by minimizing byproduct (acetate and butanediol) synthesis and a maximum of 75% enhancement in acetoin yield could be achieved. Additionally, the effect of change in ALS (acetolactate synthase) and ALDC (acetolactate decarboxylase) activities was evaluated on acetoin accumulation. Increasing ALS and ALDC enzyme activities led to efficient utilization of pyruvate towards acetoin accumulation and about 80% enhancement in acetoin accumulation was observed.

## 1. Introduction

Acetoin is a four-carbon hydroxy-keto compound that is secreted by various microorganisms when grown on glycolytic substrates. It is widely used in the food and dairy industry as a preservative [1]. Acetoin has two biological analogues, diacetyl and 2,3-butanediol. The ratio of these metabolites present depends on the culture redox potential [2].

Acetoin is a secondary carbon source synthesized during sporulation in various organisms such as* Bacillus subtilis* [3, 4],* Klebsiella terrigena *[5], and* Pelobacter carbinolicus* [6]. In* Bacillus subtilis*, acetoin synthesis involves* alsSD* operon that consists of* alsS *and* alsD *genes encoding for acetolactate synthase (ALS; E.C. 2.2.1.6) and acetolactate decarboxylase (ALDC; E.C. 4.1.1.5), respectively [3]. However, in* Klebsiella* and* Pelobacter* sp., a butanediol dehydrogenase (*bdh*) is integrated with* alsSD* operon that leads to production of 2,3-butanediol as major product instead of acetoin. Therefore,* Bacillus subtilis* is used as a model organism for study of acetoin synthesis.

A number of factors can affect the fate of metabolites by diverse alternative metabolic pathways [7]. Among them, initial substrate concentration [8, 9], temperature [10], pH [11], and oxygen levels [12] are known to affect secondary metabolites production in* Klebsiella* and* Pelobacter *sp. To our knowledge, no report exists on the assessment of these physiological factors and activities of acetoin synthesis enzymes (ALS and ALDC) for acetoin accumulation by* B. subtilis*.

Our current interest focuses on identification of important variables to enhance acetoin accumulation and minimizing byproduct formation. For this purpose, we focused on two effects. First, we focus on the systematic evaluation of physiological factors (initial substrate concentration, temperature, pH, and aeration) on growth and acetoin production. Second, we investigated the effect of ALS and ALDC enzyme activities on metabolites (glucose, pyruvate, acetate, acetoin, and 2,3-butanediol) accumulation patterns.

## 2. Materials and Methods

### 2.1. Media, Growth Conditions, and Maintenance

A list of strains with their genotype description is provided in Table 1.* B. subtilis* and* E. coli* cultures were grown in Luria Bertani (Hi Media Laboratories Pvt. Ltd., Mumbai, India) medium at 37°C and shaking at 200 rpm.* Bacillus subtilis* was also grown in Tris base medium (TSS) [13]. Unless stated, 10 g/L of glucose was used for growth studies. When required, ampicillin (100 *μ*g/mL) and chloramphenicol (25 *μ*g/mL) were used for selection of* E. coli* strains (Sigma Chemical Co., USA). Chloramphenicol (5 *μ*g/mL) was used for selection of* Bacillus *strains.

Unless otherwise mentioned, all chemicals were purchased from Sisco Research Laboratory Pvt. Ltd., Mumbai, India. Standard cloning protocols were followed in* Escherichia coli* and* Bacillus subtilis*, as described in Sambrook et al., 1989, and Harwood and Cutting, 1990, respectively.

### 2.2. Acetolactate Synthase Assay

For preparation of crude lysates, a single colony of BS7431 and BS7432 was transferred into 10 mL LB with appropriate antibiotic and incubated at 37°C for 16 hrs. 1 mL of this culture was transferred into 100 mL of LB in a 500 mL flask and was incubated at 37°C till an OD_600_ ~ 0.1. From each flask, 10 mL of the culture was aliquoted into each of 6 test tubes. In three of these tubes cultures were induced using 3 mM IPTG; the rest were used as control. After 6 hrs of incubation at 37°C, cells were pelleted down by centrifugation at 16000 g for 5 min (Hettich Zentrifugen, Micro 220R). Pellets were dissolved in a buffer containing 0.05 M HEPES (Sigma Chemical Co., USA) and 0.1 M KCl. For lysis, 1 mg of lysozyme and 10 *μ*L of protease inhibitor cocktail (Fermentas, USA) were then added and the mixture was incubated for 1 hr at 4°C. To rupture cells, 15–20 cycles of sonication with 30 seconds pulses (at 30-second intervals) were performed (Branson, Model 250). Sonicated samples were then centrifuged for 45 min at 16000 g to remove cell debris. For storage, 25% of glycerol was added to the supernatant.

The ALS activity assay was performed as per Holtzclaw et al., 1975, with some modifications. The reaction mixture for this assay contained 100 mM of K_2_HPO_4_ pH 7.0, 20 *μ*M potassium pyruvate (Sigma Chemical Co., USA), 10 *μ*M MgCl_2_, and 80 *μ*g/mL TPP (Himedia Laboratories Pvt. Ltd., Mumbai, India). The reaction was initiated on addition of 0.1 mL crude lysate and incubated for 15 min at 37°C. The reaction was terminated with 0.1 mL of 8 N H_2_SO_4_ (E. Merck, India). As a control, the crude lysate was added after the addition of H_2_SO_4_. The reaction mixtures were centrifuged for 25 min at 16000 g to precipitate the protein. The supernatant was incubated for 30 min at 37°C. 0.5 mL of supernatant was transferred to 0.1 M creatine and *α*-naphthol solution [15]. The reaction mixture was then incubated at 37°C for 10 min and the absorbance was measured at 540 nm. An enzyme unit is a measure of synthesis of 1 mole of acetoin. Specific activity of ALS was measured as the enzyme activity per milligram of total protein.

### 2.3. Cultivation Experiments

The wild-type and recombinant strains of* Bacillus subtilis* 168 were inoculated in LB (10 mL) and grown overnight at 37°C and 200 rpm. 1 mL of this overnight culture was pelleted down and washed with 0.1 M phosphate buffer saline. This washed pellet was used to inoculate 100 mL of TSS medium containing the desired carbon concentration and pH conditions as required for specific experiments. Samples for measurement of OD and metabolite were taken at 5 hr intervals until the glucose was exhausted and utilization of acetoin had started. Where required, cells were induced on addition of 3 mM IPTG (Fermentas, USA) at OD_600_ ~ 0.2. Metabolite concentrations were measured immediately without storing samples.

Estimation of glucose, pyruvate, acetate, acetoin, and 2,3-butanediol concentrations was carried out on an HPLC using Bio-Rad Aminex HPX-87H ion exclusion column of 300 mm × 7.8 mm. 5 mM H_2_SO_4_ was used as the mobile phase and column temperature was maintained at 65°C.

The results presented in this work are from 3–5 independent experiments, performed in triplicate.

## 3. Results and Discussions

### 3.1. Identification of Optimal Glucose Levels

The glucose levels present during growth of* B. subtilis* are thought to affect acetoin synthesis in two ways. First, an increase in glucose concentration leads to an increase in glycolytic flux towards acetoin synthesis and consequently an increase in acetoin accumulation may be observed [8, 9]. Second, acetoin synthesis is under catabolite repression [16, 17], and consequently a high glucose concentration (0.5%) represses acetoin synthesis [16]. Therefore, it is important to optimize the glucose concentration for efficient acetoin accumulation. Towards this objective,* Bacillus subtilis* 168 was grown in TSS media provided with 2.5, 5.0, 10.0, 20.0, 50.0, and 100.0 g/L of glucose and acetoin synthesis was measured during the course of growth.

On changing the glucose concentration from 2.5 g/L to 100 g/L, a nonlinear increase in acetoin synthesis (from 0.05 to 2.7 g/L) was observed. A maximum acetoin yield of ~0.08 mole/mole was observed for 5 and 10 g/L glucose addition. On the contrary, a decrease in acetoin yield to 0.05 mole/mole was observed on using 50 and 100 g/L of glucose. An increase in glucose concentration (10–100 g/L) results in an increase in pyruvate accumulation from 57 to 3500 mg/L. Moreover, an increase in glucose concentration also leads to shifts in time of maximum acetoin accumulation from 25 to 156 hr. The accumulation of pyruvate, residual glucose, and shift into the time point corresponding to maximum acetoin yield all suggest that efficiency of acetoin synthesis process decreases from glucose condition 10 g/L onwards (Table 2). A 10 g/L glucose level ensures an optimum acetoin yield and further studies have been performed at this level of carbon source.

### 3.2. Effect of Temperature

The effect of temperature on growth and secondary metabolite production is not universal. Synthesis of antibiotic production is highly dependent on temperature in* Streptomyces *sp. [18]. Contrariwise, in* Bacillus licheniformis*, acetoin synthesis is not dependent on temperature [10]. However,* B. subtilis* growth is affected by temperature. The influence of temperature on acetoin production by* B. subtilis* is yet not reported.* B. subtilis* was therefore grown at various temperatures (25, 30, 37, and 42°C) and acetoin accumulation and growth were estimated keeping other culture conditions constant.

**Figure 1:**
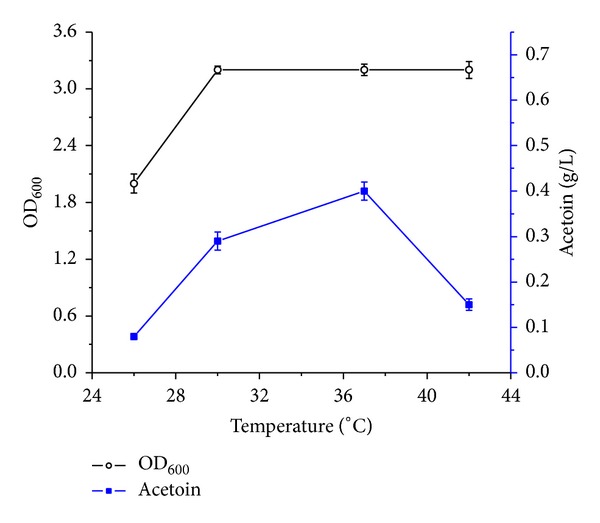
Effect of cultivation temperature on growth and acetoin synthesis by* Bacillus subtilis*.

An average cell density of OD_600_ ~ 3.2 was observed at 30, 37, and 42°C. However, a substantial (~37%) reduction of growth was observed at 25°C. Maximum acetoin accumulation was observed to be 0.41 g/L at 37°C. However, in comparison to 37°C, 70, 60, and 40% reduction in acetoin accumulation were observed at 25, 30, and 42°C, respectively (Figure 1). Variation in acetoin concentration by changing the cultivation temperature suggests that acetoin synthesis is temperature dependent in* B. subtilis*.

### 3.3. Effect of pH

An external pH shift in turn affects the transcription profile of genes globally and acidic cultivation condition leads to the induction of* alsSD* in* B. subtilis* [19, 20]. However, effect of external pH for acetoin accumulation has not been reported yet. To elucidate the effect of pH on acetoin synthesis,* Bacillus subtilis* was grown in minimal medium at various pH values from 4.5 to 9.5.* Bacillus subtilis* could not survive acidic pH of 4.5. A comparable growth of OD_600_ ~ 3.3 at pH 5.5–9.5 suggests that pH does not affect the growth of organism (data not shown).

**Figure 2:**
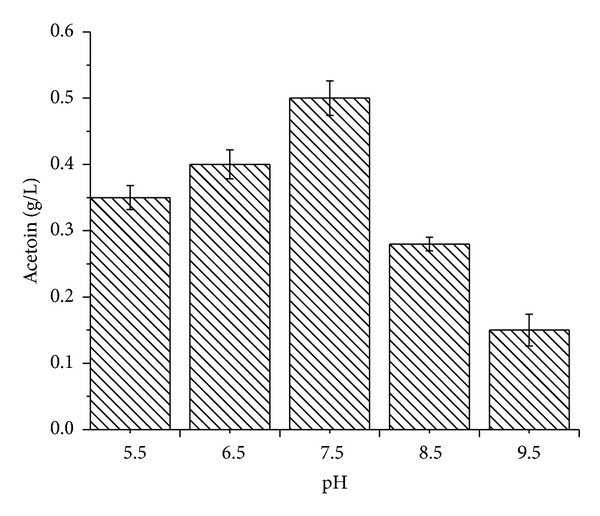
Effect of change of pH (5.5, 6.5, 7.5, 8.5, and 9.5) conditions on acetoin synthesis by* Bacillus subtilis*.

A maximum of 0.42 g/L of acetoin was observed at pH 7.5. In comparison to pH 7.5, a 20% decrease was observed at acidic pH (5.5 and 6.5). However, 20% and 75% decrease in acetoin accumulation were observed at basic pH 8.5 and 9.5, respectively (Figure 2). A lower acetoin accumulation in basic medium is due to expression of acetoin catabolic genes in alkaline conditions [20].

So, recommended pH for acetoin accumulation is 7.5 that favors both biomass and acetoin accumulation.

### 3.4. Effect of Redox Status

A higher acetoin accumulation is the function of shift of metabolite flux from glucose to acetoin and decrease in acetoin utilization. As indicated earlier, acetoin is either converted to its biological analogues diacetyl and 2,3-butanediol or catabolized to synthesize acetate; the culture redox potential is important for both reactions [21]. Redox status of culture can be changed by varying the agitation speed.* Bacillus subtilis* therefore was grown at 50, 100, 150, 200, and 250 rpm.

**Figure 3:**
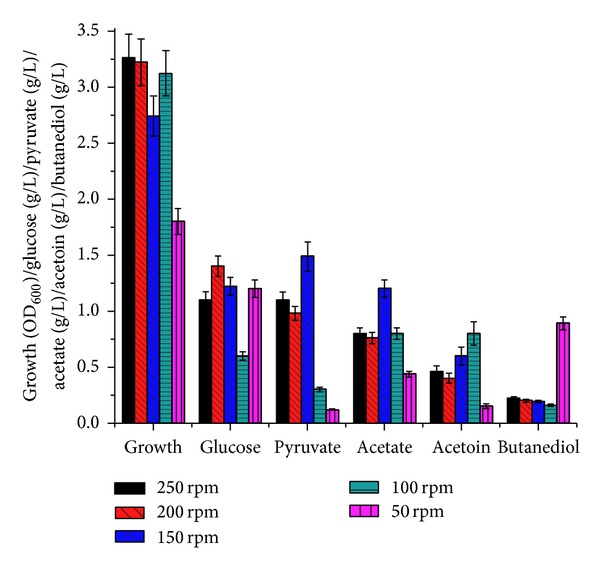
Effect of change in aeration (50, 100, 150, 200, and 250 rpm) on biomass (OD_600_), glucose, pyruvate, acetate, acetoin, and 2,3-butanediol. Data presented here is at maximum acetoin synthesis.

As expected, change in agitation speed is reflected in biomass production (3.2 and 1.7 at 250 and 50, resp.). Acetoin accumulation was observed to be maximal at 100 rpm with a 50% decrease in 2,3-butanediol and 30% reduction in acetate synthesis, in comparison to other rpm conditions (Figure 3). An agitation rate of 100 rpm is therefore optimal for acetoin accumulation.

### 3.5. Effect of Acetoin Synthesis Enzyme Activities (ALS and ALDC)

Towards understanding the effect of levels of ALS and ALDC enzymes on acetoin biosynthesis, metabolite patterns were compared for* Bacillus subtilis* 168, strains lacking* alsSD* enzymes activities (deletion of* alsR* [14]), and strains having increased* alsSD* enzyme activities.

To confirm inducible expression of* alsSD* under IPTG control, ALS and ALDC activities were compared for BS7431 and BS7432 in presence and absence of IPTG [22]. A 6.3-fold increase in specific enzyme activity was observed on addition of IPTG at 46 hr for BS7432 in comparison to BS7431. That suggests successful overexpression of* alsSD* operon under IPTG inducible control (Table 3).

**Figure 4:**
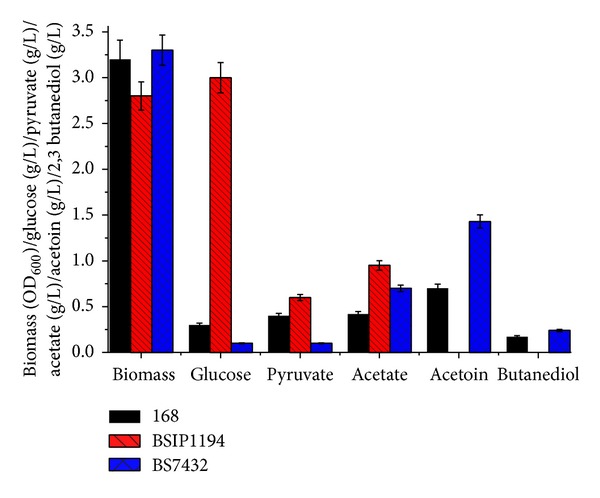
Comparative measurement for change in biomass, glucose, pyruvate, acetate, acetoin, and 2,3-butanediol for 168, BSIP1194, and BS7432. Data presented here is at maximum acetoin synthesis.

A comparable OD_600_ of 3.1, 2.9, and 3.2 was observed for 168, BSIP1194, and BS7432 that suggest that level of ALS activities does not likely have an effect on viability of cells. In absence of ALS activity, 2.9 g/L of glucose and 0.5 g/L pyruvate were observed. However, none is observed for 168 and BS7432 that suggests that levels of ALS affect glucose and pyruvate assimilation. Probable reason for residual glucose and accumulated pyruvate is inefficient metabolism in absence of functional* alsSD*. A 30% enhancement in acetate accumulation (Figure 4) was observed in BSIP1194 that could be a result of shift of major pyruvate towards acetate synthesis in absence of acetoin biosynthetic pathway.

As acetate and 2,3-butanediol are the products of acetoin catabolism, an increase of acetoin biosynthesis is reflected in 50% increase in acetate and 40% increase in acetate (Figure 4). Increase of ALS and ALDC enzyme activities causes an 80% increase in accumulation of acetoin (0.5 of 168 and 1.2 in BS7432).

**Figure 5:**
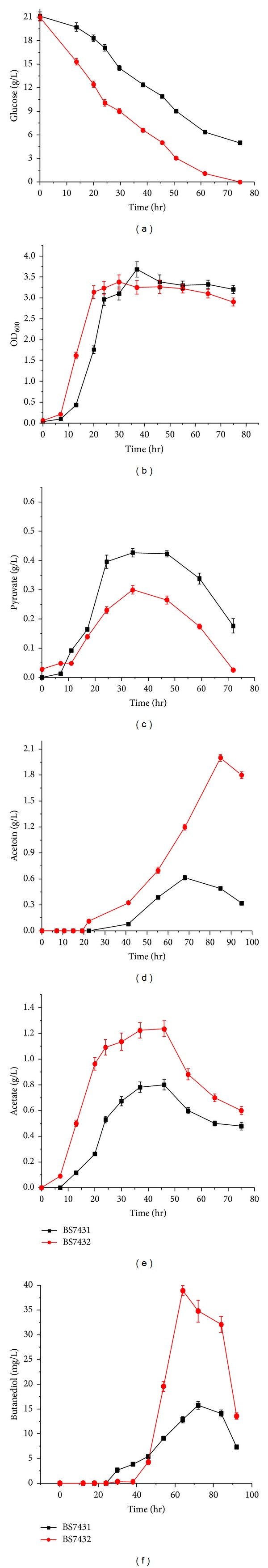
Comparative measurement of (a) biomass, (b) glucose, (c) pyruvate, (d) acetate, (e) acetoin, and (f) 2,3-butanediol for BS7432 with BS7431 at pH 7.5 by BS7431 and BS7432.

As only 10 mg/L of pyruvate was observed at maximum acetoin biosynthesis in BS7432, the reason of increased acetoin synthesis could be due to efficient utilization of pyruvate towards acetoin synthesis [23]. To reconfirm this, BS7431 and BS7432 were grown in medium conditions where residual glucose and accumulated pyruvate remain in growth medium (TSS + 20 g/L) and change in glucose (Figure 5(a)), biomass (Figure 5(b)), pyruvate (Figure 5(c)), acetoin (Figure 5(d)), acetate (Figure 5(e)), and 2,3-butanediol (Figure 5(f)) was measured.

Expression of* alsSD* did not cause any change on growth (Figure 5(b)). There was no residual glucose which remains in the medium (Figure 5(a)). Additionally, in opposition to BS7431, in BS7432, all the pyruvate got exhausted by the end of 70 hr. These results strengthen the hypothesis of involvement of* alsSD* in efficient utilization of pyruvate and glucose towards acetoin synthesis. A maximum of 1.9 g/L of acetoin accumulation was observed due to shift of flux from unused glucose and pyruvate (Figure 5(c)) [23]. Interestingly, expression of* alsSD* caused a 30% elevation in acetate level. Probably, elevated level is the product of acetoin catabolism.

## 4. Conclusions

The effect of glucose concentration, temperature, pH, agitation, and enzyme amount was investigated on acetoin synthesis by* Bacillus subtilis* 168 in minimal medium. Growth of* Bacillus subtilis* is affected by pH, temperature, and agitation. However, synthesis of byproducts (acetate and 2,3-butanediol) depends on agitation and pH of cultivation medium. By optimizing physiological conditions (glucose concentration, pH, temperature, and agitation), a maximum of 75% enhancement in acetoin yield was achieved (Table 4).

A maximum of 80% enhancement in acetoin accumulation was achieved by increasing ALS and ALDC enzyme activities. Increase in these enzyme activities is affected by efficient utilization of pyruvate towards acetoin synthesis (Table 4).

Comparable amplitude of change in acetoin yields by physiological variables and genotype changes suggests that optimal physiological conditions are equally important as that of genotype constituent.

Based on these results, we conclude that, to maximize acetoin accumulation, it is important to control physiological variables as well as genotype constituents to ensure efficient pyruvate metabolism towards acetoin.

This methodology thus can become a guideline and useful tool to the large scientific and industrial communities that are developing methods for accumulating acetoin.

## Figures and Tables

**Table 1:** List of plasmid, strains, and their sources.

Plasmids/strain	Genotype description	Source
*pHCMC *	*E. coli, Bacillus* shuttle vector, Cam^r^, Ap^r^	BGSC*
*pHCMC-alsSD *	*alsSD* (2.5 Kb) operon cloned at *BamH1* and *XbaI* site of pHCMC05	Lab stock
*Bacillus subtilis* 168	trpC2	BGSC*
BS7431	trpC2 pHCMC	Lab stock
BS7432	trpC2 pHCMalsSD	Lab stock
BSIP1194	trpC2 alsR::cam	[14]

*Bacillus Genetic Stock Center.

**Table 2:** Variation in titers of pyruvate and acetoin by change of initial glucose concentration.

Glucose (g/L)	Time of maximum acetoin yield (h)	Maximum acetoin* (g/L)	Pyruvate* (mg/L)	Residual glucose at maximum acetoin yield* (g/L)	Yield (mol·mol^−1^)*
2.5	28	0.08 ± 0.06	5	0.6 ± 0.09	0.06
5	40	0.2 ± 0.03	50	0.8 ± 0.11	0.08
10	40	0.4 ± 0.08	58	3 ± 0.08	0.08
20	75	0.71 ± 0.05	172	5 ± 0.26	0.07
50	125	1.4 ± 0.10	2800	12 ± 1.26	0.05
100	156	2.7 ± 0.12	3500	23 ± 1.96	0.05

*Time of maximum acetoin accumulation.

**Table 3:** Comparison of ALS enzyme activities of BS7431 and BS7432 under IPTG induced and uninduced condition.

Serial number	Sample name	Average enzyme activity (formation of 1 nmol of acetolactate per minute)	Specific enzyme activity (unit/mg of protein)
1	BS7431-Un*	3.6 ± 0.16	0.52 ± 0.06
2	BS7431-In^#^	4.50 ± 0.12	0.64 ± 0.08
3	BS7432-Un*	6.75 ± 0.12	0.95 ± 0.02
4	BS7432-In^#^	34.5 ± 0.16	4.86 ± 0.02

*Uninduced cells; ^#^induced cells.

**Table 4:** Comparison of acetoin yield, specific acetoin production, and maximum possible change in acetoin accumulation by variation of glucose, pyruvate, temperature, pH, aeration, and acetoin synthesis enzyme.

Physiological parameters	Acetoin yield (mole of acetoin produced/mole of glucose used)	% maximum possible change in acetoin accumulation
Glucose (g/L)		
2.5	0.004	32.0
5	0.02	
10	0.081	
Temperature		
25	0.020	30.1
37	0.101	
42	0.024	
pH		
5.5	0.071	38.5
7.5	0.108	
9.5	0.051	
Aeration (rpm)		
50	0.040	49.8
100	0.143	
250	0.102	
ALS and ALDC		
168	0.107	80.3
BS7432	0.245	

*Percentage of the highest acetoin accumulation is calculated as {highest acetoin titre (g/L) − lowest acetoin titre (g/L)/highest acetoin titre (g/L)}∗100.
